# Association of Social Determinants of Health and Road Traffic Deaths: A Systematic Review

**DOI:** 10.30476/beat.2020.86574

**Published:** 2020-10

**Authors:** Mina Saeednejad, Farideh Sadeghian, Mahsa Fayaz, Dennis Rafael, Rasha Atlasi, Amirmasoud Kazemzadeh Houjaghan, Raziyeh Abedi kichi, Mohammad Hossein Asgardoon, Hossein Zabihi Mahmoudabadi, Zahra Salamati, Zohrehsadat Naji, Vafa Rahimi-Movaghar, Payman Salamati

**Affiliations:** 1 *Sina Trauma and Surgery Research Center, Tehran University of Medical Sciences, Tehran, Iran*; 2 *Center for Health Related Social and Behavioral Sciences Research, Shahroud University of Medical Sciences, Shahroud, Iran*; 3 *Department of Epidemiology and Biostatistics, School of Public Health, Shahroud University of Medical Sciences, Shahroud, Iran*; 4 *Faculty of Health - School of Health Policy & Management, University of Toronto, Toronto, Canada *; 5 *Department of Medical Library and Information Science, School of Allied Medical Sciences, Tehran University of Medical Sciences, Tehran, Iran*; 6 *Sina hospital, Department of surgery, school of medicine, Tehran University of medical sciences, Tehran, Iran*; 7 *School of Architecture, College of Fine Arts, University of Tehran,* *Tehran, Iran*; 8 *Young Researchers and Elites Club, Science and Research Branch, Islamic Azad University, Tehran, Iran*

**Keywords:** Social Determinants of Health, Accidents, Traffic

## Abstract

**Objective:**

This study aims to review systematically the association of social determinants of health (SDH) and road traffic deaths (RTD) within scientific literature.

**Methods:**

A search strategy was designed and run in EMBASE, PubMed via MEDLINE, Scopus, Web of Science, and Cochrane library. Through title, abstract, and full-text screening, all English original papers (except ecological studies) which studied social determinants of health and fatal injuries were included. Papers which studied association between RTD and the education, income, rural settlement, and marital status were evaluated and the related data was extracted from the full-texts.

**Results:**

Eleven articles out of 7,897 primary results were selected to be included in the study. Among eight papers studied education, seven confirmed a negative association between years of schooling and RTD. Two out of three articles reported no association between income leveland RTD. Among three papers studied rural settlement, two approved a positive relationship between this determinant and RTD. Both articles studied marital status, confirmed an association between this determinant and RTD.

**Conclusion:**

A few papers studied association of social determinants of health (SDH) and RTD. There was an inverse relationship between education and RTD. The evidence for such an association between income, rural settlement, and marital state was scarce. Further investigations are recommended through original research.

## Introduction

Every year, about 20 to 50 million people are injured [1] and injuries cause about five million deaths worldwide [2] which contribute eight percent of all deaths globally [3]. Forty years ago, Haddon presented a new matrix for analyzing causes of injuries in which there were two dimensions of factors and phases [4]. He categorized factors into four items including human, vector (vehicle), physical, and social environments and phases into three stages including pre-event, event, and post-event. For example, in road traffic crashes (RTC), societal factors could be considered as cultural norms permitting speeding for pre-event phase, lack of vehicle design regulation for event phase, and lack of support for EMS (emergency medical service)  and trauma systems for post-event phase, respectively. Although Haddon created a new perspective  on  injury analysis and attracted much attention in scientific literature, the matrix did not well noticed to social determinants of health (SDH). These determinants  are mainly shaped by economics, social policies, and politics. «They are circumstances in which people are born, grow up, live, work, and age » [5]. The SDH are related to road traffic  injuries (RTI) through a variety of pathways such as education, income, rural and urban settlement, marital status, employment, and housing [6]. In other words, the link between socioeconomic status (SES) and RTI is mediated by conditions in family, neighborhood, housing, workplace, etc.

Several studies confirm that RTIs have been decreased in recent decades across the globe [7]. Different studies showed that declines in SES are associated with rising in fatal and non-fatal injuries and vice versa  [8-11]. The Canadian Institute for Health Information reported that the frequency of injury was 1.3 times higher among the poorest Canadians compared to the wealthiest [12]. Indeed, poverty is linked to a sense of insecurity, hopelessness and a lack of resources and opportunities  [13] and jeopardizes health generally [14]. Despite there are many published papers about SDH, articles regarding the social determinants of road traffic deaths (RTD) are scarce. The aim of this study was to present a valid research, through a systematic review of related resources to determine social determinants of RTD.

## Materials and Methods

*Information resources*

Electronic resources and databases including EMBASE, PubMed via MEDLINE, Scopus, Web of Science, and Cochrane library were comprehensively and systemically searched for inclusion of papers published in international journals. The date of search in the scientific databases carried out on 9 June 2019  and all documents published and indexed in the searched databases, until this date, had retrived.

*Search strategies*

In this systematic review, we conducted the search strategy for each databases according to their features and capabilities and retrieved all of the documents that were indexed in the above mentioned databases. We designed  the search strategies by using the main concepts which included injury, fatal  and sociological factors and their synonyms and related terms. then these terms and phrases applied for search in the title, abstract and keywords of the documents.  No limits such as language or date or document type in this seach strategy applied to find all of the related document as possible. The search strategy for the PubMed  searchengine databases was as follow:

("Social Determinants of Health"[Mesh]  OR  "Sociological Factors"[Mesh]  OR  ((Sociological[TIAB]  OR   Social [TIAB] OR Socioeconomic[TIAB]) AND    (Determinant [TIAB]  OR Determinants[TIAB]   OR determining [TIAB] OR Factor [TIAB] OR Factors[TIAB] OR Characteristic*[TIAB]  )) OR Inequality[TIAB] OR Inequalities[TIAB]   OR (Living [TIAB] AND Standard [TIAB]  ) OR (Land[TIAB]   AND Tenure[TIAB]  ) OR (  High[TIAB]   AND Income[TIAB]   AND Population* [TIAB]   )   )

AND

("Fatal Outcome"[Mesh]  OR Fatal* [TIAB]   OR death [TIAB]   OR dead  [TIAB]  OR deadly [TIAB]    OR Mortal [TIAB] OR Mortality[TIAB]       )

AND

("Wounds and Injuries"[Mesh] OR Injur*[TIAB]    OR Trauma*[TIAB]   OR Wound [TIAB]  OR Wounds[TIAB]   )

*Study selection, data extraction, and quality of the evidence*

All of the English original papers which studied probable social risk factors of fatal injuries were included, so studies designed as reviews, case reports, case series, and other non-original papers were not included. Moreover, ecological studies were excluded to allow for more careful identification of the social determinants of health injures. The records were reviewed by six trained independent researchers in three paired groups and screened at two levels (title and abstract screenings). Papers, were included when both of the researchers in each group were in agreement. Disagreements about any paper were resolved through discussion with a third colleague. In the next step, papers pertaining of RTD were selected and those with determinants for which more than one paper was found were reviewed.    These included determinants such as education, income, rural settlement,  and marital status. The researchers extracted the data and completed a checklist which was devised for assessing quality of studies and related variables comprised: first author, published year, name of journal, type of study, location of study, and number of participants.  

## Results

**Fig. 1:**
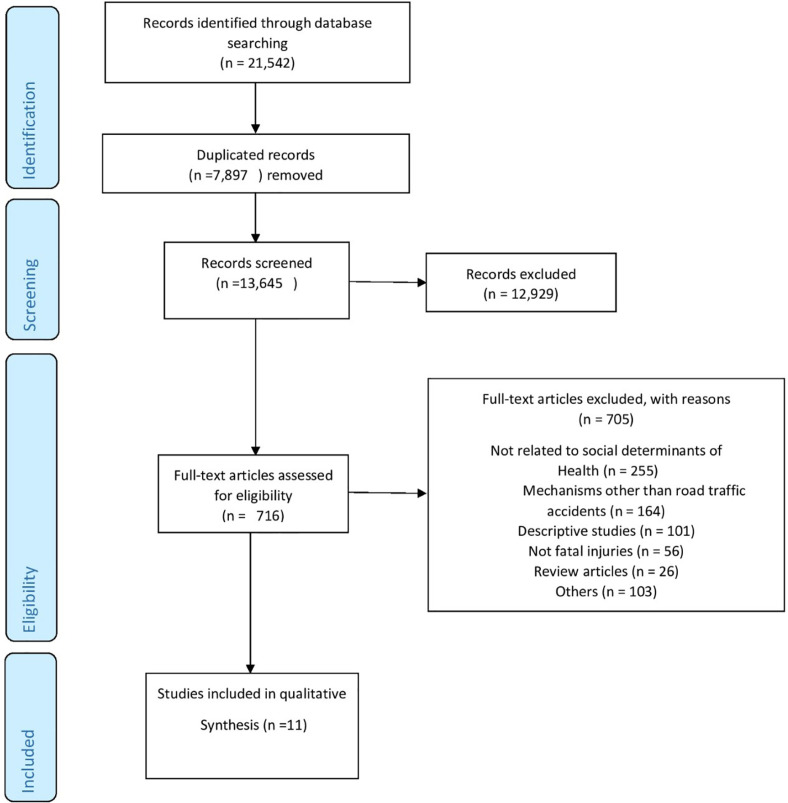
Screening of the studies based on PRISMA statements

In the initial search, 21,542 documents were obtained in which 7,897 duplicate records were deleted, leaving 13,645 for title and abstract screening. Of these records, 12,929 irrelevant records were excluded through the title and abstract screening and 716 records were screened for full-text screening in which 705 records were excluded. Finally, Eleven articles out of  7,897 primary results were selected to be included in the study (Figure 1). Eight papers studied education, three articles studied income, three papers studied rural settlement and two articles studied marital status.

*Education*

Seven papers discussed the relationship between RTD and education (Table 1). Singh *et al*. compared cause-specific mortality between the US-born population and immigrants for more than 25 years [15]. Their data indicated estimated hazard ratios or relative risk for different educational levels among those killed in motor vehicle crashes. They found a negative relationship between the years of education and the RTD in men but not in women. For men, using 15 years or more education as a reference level, the risk was 1.85 for 0-8 years of education, 2.11 for 9-11 years of education, and 1.83 for 12 years of education (*p* value < 0.05).

**Table 1:** The characteristics of the papers which evaluated the relationship between road traffic deaths and education

**First author**	**Published year**	**Journal**	**Type of study**	**Location of study**	**Number of participants**
**Singh**	2001	American Journal of Public Health	Cohort	The United States	141,323 men & 159,860 women
**Spoerri**	2011	Accident Analysis & Prevention	Cohort	Switzerland	5.5 million residents aged 18-94 years
**Barreto**	1997	International Journal of Epidemiology	Nested case control	Brazil	21,816 steel workers
**Arroyave**	2014	Preventive Medicine	Cross-sectional	Colombia	633,906 deceased people
**Braver**	2003	Accident Analysis & Prevention	Cross-sectional	The United States	15,839 deaths for 25-64-year old people
**Harper**	2015	American Journal of Epidemiology	Cross-sectional	The United States	Motor vehicle deaths in 1995 (26,2920) and in 2010 (23,897) among adults aged 25≤
**Tjepkema**	2012	Health Reports	Cohort	Canada	2.7 million people aged 25 or older
**Bawah**	2014	Injury Epidemiology	Cross-sectional	Ghana	7,465 adult (15-59) deaths

Spoerri *et al*. evaluated socio-demographic and geographical determinants of RTD by using census and RTA mortality records in Switzerland [16]. They found the hazard ratio (HR) of RTA-related death was higher in individuals with compulsory primary education in comparison with the persons with higher educational attainment (HR 1.53; 95% CI 1.29-1.81). The association was strongest for pedestrians (HR 1.87; 95% CI 1.2-2.91).

Barreto *et al*. conducted a nested case-control study in which the cases were steelworkers who died of road traffic crashes (RTC) during the employment period and controls were the employed workers born the year as the case [17]. They showed that the risk of RTD for workers with >8 years of education was significantly less than those with

Arroyave *et al*. evaluated educational levels among adult premature mortality (ages 25-64) for specific causes of deaths from 1998 to 2007 in Colombia [18]. They found that RTD were more common in people with primary education (Men: Rate Ratio 2.28; 95% CI 2.17-2.40 & Women: Rate Ratio 1.52; 95% CI 1.32-1.74) and secondary education (Men: Rate Ratio 1.91; 95% CI 1.82-2.01 & Women: Rate Ratio 1.26; 95% CI 1.12-1.41) compared to tertiary education.

Braver computed passenger vehicle occupant deaths per 10 million trips by socioeconomic status using 1995 data from the Fatality Analysis Reporting System and Nationwide Personal Transportation Survey in the USA [19]. They found rate ratios for those who had not completed high school were 3.52 (95% CI 3.39-3.65) for men and 2.79 (95% CI 2.69-2.91) for women. Also, they were 2.57 (95% CI 2.51-2.62) for men and 1.81 (95% CI 1.78-1.84) for women who had graduated from high school compared to those with more than high school education.

Harper *et al*. found education disparities among motor vehicle deceased in the US from 1995 to 2010 [20]. They found greater reductions in mortality rates between 1995 and 2010 among college graduates (−6.4 deaths per 100,000 populations, 95% CI −8.9- −4.0) and high school graduates (−6.5 deaths per 100,000 populations, 95% CI −8.6- −4.3). There was a widening gap between people with less than a high school diploma and all other groups. They showed a rise in adjusted rates among persons with less than a high school diploma compared to others (2.75 deaths per 100 million vehicle-miles travelled, 95% CI 0.29-5.21). 

Tjepkema *et al*. evaluated cause-specific age standardized mortality rates by education from the 1991 to 2006 Canadian census mortality follow-up study among 2.7 million people aged 25 or older [21]. For men, the mortality gradient increased by educational attainment groups step by step (university degree, post-secondary diploma, secondary graduation, and less than secondary graduation). The Rate ratio was 1.98 comparing people with less than secondary education to those with a university degree; however, the statistics were not significant for women.

Otherwise, Bawah *et al*. investigated the determinants of RTD in one of the rural districts of northern Ghana in 2014 [22]. They showed that RTD was more among people with secondary or tertiary education compared to those without any education (OR 3.21 95% CI 1.75-5.87).

*Income*

Three papers studied the relationship between RTD and income (Table 2). Tjepkema *et al*. in their another paper, reported cause-specific age standardized mortality rates by income adequacy from the 1991-to-2006 Canadian census mortality and cancer follow-up study among 2.7 million people aged 25 or older [23]. The RTD gradient decreased by income adequacy quintile (Q1 to Q5). Rate ratios were 1.45 and 1.51 for men and women, respectively (*p* value< 0.05).

**Table 2:** The characteristics of the papers which evaluated the relationship between road traffic deaths and income

**First author**	**Published year**	**Journal**	**Type of study**	**Location of study**	**Number of participants**
**Tjepkema**	2013	Health Reports	Cohort	Canada	2.7 million people aged 25 or older
**Singh**	2001	American Journal of Public Health	Cohort	The United States	141,323 men & 159,860 women
**Barreto**	1997	International Journal of Epidemiology	Nested case control	Brazil	21,816 steel workers

Also, Singh *et al*. compared cause-specific mortality between the US-born and immigrants and estimated risk indexes for different income levels [15]. They found no relationship between family income levels and the RTD in both men and women.

Similarly, as mentioned earlier, Barreto et al. found no relationship between the risk of death from motor-vehicle injury and levels of salaries among the research participants [17].

*Rural settlement*

Three papers studied the relationship between RTD and rural settlement (Table 3). Chen *et al*. evaluated RTD of young drivers aged 17-25 from the beginning of 1997 to the end of 2007 [24]. Location of residence was classified as urban, regional, or rural. They showed that fatality rate was about 2-3 times higher among rural drivers than urban drivers. Moreover, the relative risk increased from 3.6 in 1997 to 6.0 in 2007.

**Table 3:** The characteristics of the papers which evaluated the relationship between road traffic deaths and rural settlement

**First author**	**Published year**	**Journal**	**Type of study**	**Location of study**	**Number of participants**
**Chen**	2010	Journal of Safety Research	Cross-sectional	New South Wales State, Australia	644 crash fatalities for drivers aged 17-25 years old
**Kristensen**	2012	Injury Prevention	Cohort	Norway	611,654 all Norwegians born in 1967-1976 from age 16 years
**Bawah**	2014	Injury Epidemiology	Cohort	Ghana	7,465 adult (15-59) deaths

Kristensen *et al*. evaluated rural and urban settlement inequalities in RTD at age 16 to 20 years among Norwegians born from 1967 to 1976 [25]. They found no relationship between RTD and rural/urban settlement; however, by categorizing crashes as either collision (vehicle to vehicle) or non-collision, they found male non-collision death rates were increased in rural municipalities (Rate Ratio 1.66 95% CI 1.20-2.29).

Bawah *et al*. studied determinants of RTD in Ghana [22]. They found that RTD was higher among urban residents in comparison to rural residents (OR 1.74 95% CI 1.09-2.78).

*Marital state*

Two papers studied the relationship between RTD and marital status (Table 4). Spoerri *et al*. examined socio-demographic and geographical determinants of RTD in Switzerland and found that the hazard ratios of RTC-related deaths were higher in divorced individuals (HR 1.62; 95% CI 1.33-1.97), singles (HR 1.24; 95% CI 1.05-1.46), and widowed persons (HR 1.31; 95% CI 1.05-1.65) in comparison with those who were married [16].

**Table 4:** The characteristics of the papers which evaluated the relationship between road traffic deaths and marital state

**First author**	**Published year**	**Journal**	**Type of study**	**Location of study**	**Number of participants**
**Spoerri**	2011	Accident Analysis and Prevention	Cohort	Switzerland	5.5 million residents aged 18-94 years
**Barreto**	1997	International Journal of Epidemiology	Nested case control	Brazil	21,816 steel workers

Barreto *et al*. study showed the risk of death from motor-vehicle injury was independently associated with being unmarried (OR 3.21; 95% CI 1.84-5.59) compared to being married [17].

## Discussion

In this research, only the determinants for which more than one paper had been found in the databases were included in the study. As a result, four social determinants of health (SDH) including education, income, rural settlement, and marital state were evaluated in the paper. Considering education variable, seven out of eight papers found an inverse significant relationship between this factor and RTD. Only the Bawah’s study showed a different finding. As the study conducted in a rural district of Ghana (an African country), the participants might be from a lower SES and some of them could not afford motor vehicles, so they were not a good representative of general population.

Furthermore, four of the papers compared the mortality in accordance of gender. Although Arroyave and Braver reported such a relationship in both males and females, Singh and Tjepkema did not find any difference.

Moreover, such a relationship was found between education and all external causes of deaths through other studies. Singh *et al*. conducted another cohort study using the National Vital Statistics System and the National Longitudinal Mortality Study in the USA [26]. They studied trends and differentials in adolescent and young adult mortality from 1950 to 1993. They showed that education was negatively related to fatal injuries. Specifically, young men aged 20 to 24 years with 8 or fewer years of education had a 160% higher mortality risk compared to those with 13 or more years of education.

In a nested case-control study, Barreto *et al*. evaluated steelworkers who worked in a Brazilian steel plant in which the cases died of any injuries during the employment period and the controls were the employed workers born the year as the case [27]. They showed that the years of schooling was inversely associated with the risk of fatal injuries (*p*=0.03). Meanwhile, men with less than five years of education and those with five to eight years of education had four times the risk than men with nine or more years of education.

Considering income variable, although Tjepkema found a negative relatonship between RTD and income level in both men and women, Singh and Barreto did not confirm this idea.

In an ecological study, Elmen *et al*. evaluated childhood, youth, and early adulthood RTD in the city of Goteborg, Sweden from 1971 to 1985 [28]. They divided citizens into three socio-economic area groups according to income and assessed major causes of deaths among them. They showed that age adjusted mortality rate was more among men with low/medium income level compared to those with high income level both in 15-24 year and 25-44 year groups (*p*p<0.001, respectively). Also, in 1991, Van Beeck *et al*. conducted an ecological study and evaluated all RTD from 1980 to 1984 in Netherland  [29]. They found a highly significant inverse association between RTD and per capita income.

Considering rural settlement variable, depite Chen and Kristensen found a positive relationship between rural settlement and RTD, Bawah reported a completely different finding indicating such relationship between urban settlement and RTD. As pointed earlier, the socioeconomic status of participants in Bawah’s study was not a good representative of general population.

Using the Texas Healthcare Information Council database in the US, Culica et al evaluated all fatal injuries who occurred among patients hospitalized in the state from 1999 to 2000 [30]. They found that in rural areas, mortality was higher among the following groups: adults 25-44 years old, Hispanics, uninsured patients, and those admitted through transfer. In urban areas, mortality was higher among the following groups: adults 18-24 years old and those admitted as severe emergencies.

In a case control study, Ziraba *et al*. evaluated causes and risk factors of fatal injuries in two slums of Nairobi city in Kenya from 2003 to 2005 [31]. With controlling covariates, they showed that only areas in which more than 75% of households were single-member had a significantly higher risk of fatal injuries. 

Considering marital state variable, both Spoerri and Barreto confirmed a relationship between RTD and marriage. Likewise, Spoerri found that among unmarried persons, the risk decreased in the order of divorced, singles, and widowed persons, respectively.

Singh et al. in their cohort showed such a relationship between marriage and all external causes of deaths in 1996. They found that divorced, separated, and widowed persons had a 2.2 times higher risk of mortality than married subjects [26]. Fauveau *et al*. evaluated women aged 15-44 years old living in a sub-district of rural Bangladesh from 1976 to 1986 [32]. They showed that unmarried girls had 2.3 times more fatal injuries than married women (relative risk 1.7; 95% CI: 1.3-2.3).

Furthermore, findings that social determinant of health are relevant to understanding road traffic deaths raises the question of how to respond to these findings.  While mid-level interventions such as greater traffic regulation enforcement and educational campaigns will always be present, it may be that social determinants of health are a fundamental cause of such deaths [33, 34]. As such, reducing road traffic deaths may require systematically attempting to improve the quality and equitable distribution of the social determinants of health through public policy action. This was the view taken by the WHO Commission on Social Determinants of Health [35]. Such public policy action will be more likely in nations who see a State role as promoting equality and equitable distribution of economic and social resources [36]. Indeed, there is evidence that road traffic fatalities are higher in nations with greater overall income inequality, suggesting that broader macro-level indicators of road traffic fatalities are a fruitful area for further inquiry [37].

This study had a few limitations. Ecological studies and non-original papers like reviews, case reports, and case series are not indicated to be included. Likewise, only English articles were reviewed. As a result, they might affect data extraction process and could shorten the result section.

In conclusion, scientific literature about SDH is scarce. Although it showed that there was a negative relationship between education and RTD, a few articles studied the association for income, rural settlement, and marital state. Further original studies are needed for better decision making.
